# Identification of a WRKY transcriptional activator from *Camellia sinensis* that regulates methylated EGCG biosynthesis

**DOI:** 10.1093/hr/uhac024

**Published:** 2022-02-19

**Authors:** Yong Luo, Xiang-xiang Huang, Xiao-feng Song, Bei-bei Wen, Nian-ci Xie, Kun-bo Wang, Jian-an Huang, Zhong-hua Liu

**Affiliations:** 1 School of Chemistry and Environmental Science, Xiangnan University, Chenzhou, Hunan, 423000, China; 2 Key Laboratory of Tea Science of Ministry of Education & National Research Center of Engineering and Technology for Utilization of Botanical Functional Ingredients, Hunan Agricultural University, Changsha, 410128, China; 3 College of Tea Science, Guizhou University, Guiyang, 550025

## Abstract

Naturally occurring methylated catechins, especially methylated EGCG in tea leaves, are known to have many health benefits. Although the genes involved in methylated EGCG biosynthesis have been studied extensively, the transcription factors that control methylated EGCG biosynthesis are still poorly understood. In the present study, a WRKY domain-containing protein termed CsWRKY57like was identified, which belongs to group IIc of the WRKY family and contains one conserved WRKY motif. CsWRKY57like was found to localize in the nucleus and function as a transcriptional activator; its expression was positively correlated with methylated EGCG level. In addition, CsWRKY57like activated the transcription of three genes related to methylated EGCG biosynthesis (*CCoAOMT*, *CsLAR*, and *CsDFR*), specifically interacting with their promoters by binding to the *cis*-acting element (C/T)TGAC(T/C). Further assays revealed that CsWRKY57like physically interacts with CsVQ4 and participates in the metabolic regulation of *O*-methylated catechin biosynthesis. We conclude that *CsWRKY57like* may positively impact the biosynthesis of methylated EGCG in the tea plant. These results comprehensively enrich the regulatory network of WRKY TFs associated with methylated EGCG and provide a potential strategy for the breeding of specific tea plant cultivars with high methylated EGCG levels.

## Introduction

**Figure 1:**
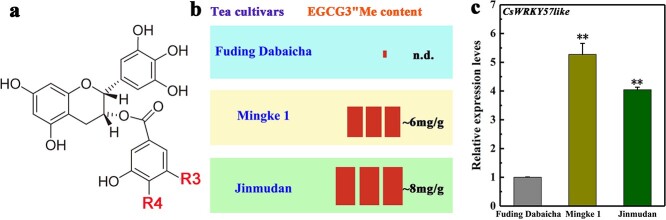
The contents of EGCG3″Me and the expression of *CsWRKY57like*. (a) The chemical structure of EGCG3″Me [7]. (b) The contents of EGCG3″Me cited by previous studies [9]. (c) The relative expression level of *CsWRKY57like* by qRT-PCR. ^**^represents a significant difference compared to ‘Fuding Dabaicha’ (*P* < 0.01).

Tea is the second most consumed beverage in the world after water. The quality of tea and its health-promoting effects on humans depend on its leaves and leaf buds, which are rich in varied secondary metabolites, especially flavonoids, theanine, and caffeine [1–3]. Numerous studies suggest that tea plays a significant role in reducing vascular disease, heart disease, and cancers [4, 5]. Catechin, especially epigallocatechin gallate (EGCG), is the main functional component in tea leaves. Recently, methylated tea catechin derivatives, especially (−)-epigallocatechin-3-*O*-(3-*O*-methyl)-gallate (EGCG3′′Me, Figure 1a), have attracted significant attention for their role in the prevention of arteriosclerosis and their strong antiallergic and antihypertensive activities [6–8]. Catechin is an important and abundant compound in tea plants and can be detected in all tea plant tissues. However, methylated catechins are present in limited quantities in a few tea cultivars. Previously, we found that EGCG3″Me accumulated to high levels in two tea cultivars, ‘Jinmudan’ and ‘Mingke 1’, among several Chinese tea cultivars [9] (Figure 1b). Nevertheless, the molecular mechanism of methylated catechin biosynthesis and the transcriptional regulatory mechanism of related genes during EGCG3′′Me production remain unclear. Therefore, an in-depth analysis of the catechin biosynthesis regulatory network is essential for breeding tea plant cultivars with high EGCG3′′Me content.

Methylated EGCG is a flavonoid, and it is biosynthesized through the phenylpropanoid and flavonoid pathways [9–11]. The structural genes involved in the biosynthesis of methylated catechins have been researched intensively, including anthocyanidin reductase (*ANR*), anthocyanidin synthase (*ANS*), leucoanthocyanidin reductase (*LAR*), dihydroflavonol-4-reductase (*DFR*), and caffeoyl-CoA O-methyltransferase (*CCoAOMT*). These different genes may play multiple roles in flavonoid and catechin biosynthesis. Chen et al. [12] hypothesize that *CsANR1*, *CsANR2*, and *CsANS* are the major regulatory genes that affect non-epicatechin formation. Our recent data showed that the expression levels of the methylated catechin biosynthetic genes *CsLAR*, *CsDFR*, and *CCoAOMT* were positively correlated with the accumulation of EGCG3′′Me [9], indicating that the biosynthesis of methylated catechins may be a complex process mediated by the dynamic balance of putative enzymes at the transcript and protein levels. However, the transcriptional regulatory mechanism of methylated catechin biosynthesis is still unclear.

Many transcription factors (TFs) are known to be involved in the biosynthesis of flavonoids in different plant species; these include members of the MYB, WRKY, bHLH, and WD40 families [13–16]. In tea plants, potential TFs controlling catechin biosynthetic pathways have been identified, but there has been little functional characterization. The WRKY TFs are a type of protein that is structurally defined by the presence of one or two WRKY domains [17]. Numerous studies indicate that WRKYs can control target gene expression via interaction with specific DNA sequences, and they are involved in plant growth and adaptation to biotic and abiotic stresses [18–20]. More importantly, several members of the WRKY TFs are involved in flavonoid biosynthesis. For example, MdWRKY11 was found to participate in the biosynthesis of flavonoids in apple (*Malus*) [14], and a grapevine TTG2-like WRKY is reported to regulate flavonoid biosynthesis and vacuolar transport [21]. Previously, we identified two WRKY TFs that may negatively affect methylated EGCG biosynthesis. However, whether WRKYs in tea plants can upregulate the biosynthesis of polyphenols, particularly methylated catechin, is not clear.

In this study, the common tea cultivar ‘Fuding Dabaicha’ and the two high-EGCG3′′Me tea cultivars ‘Jinmudan’ and ‘Mingke 1’ were used for transcriptomic analysis. A positive transcriptional activator termed CsWRKY57like was identified, which may be associated with methylated catechin biosynthesis. A dual-luciferase assay was carried out to investigate the transcriptional regulation by CsWRKY57like of promoters of three genes related to methylated EGCG biosynthesis (*CCoAOMT*, *CsLAR*, and *CsDFR*) in tobacco. ChIP-PCR and EMSA were used to evaluate the binding activities of CsWRKY57like to the promoters of *CsLAR*, *CsDFR*, and *CCoAOMT*. Our findings provide new evidence for the WRKY-mediated regulation of methylated EGCG biosynthesis in tea plants.

## Results

### FPKM expression analysis of *CsWRKY57* genes among different cultivars

WRKY TFs play an important regulatory role in plant metabolism. To further study the possible role of the *CsWRKY* genes in the biosynthesis of methylated EGCG, three tea cultivars with different methylated EGCG contents (Figure 1b, ‘Fuding Dabaicha’, ‘Mingke 1’, and ‘Jinmudan’) were subjected to transcriptomic analysis. Previous studies have identified and characterized fifty-nine WRKY genes in the tea genome (http://www.plantkingdomgdb.com/tea_tree/) [22]. We performed an FPKM statistical analysis, in which FPKM values were calculated to indicate the expression of *CsWRKY57like* [|log2(Fold Change)| > 0]. Interestingly, our results suggested that *CsWRKY57like* had higher expression in ‘Mingke 1’ and ‘Jinmudan’ cultivars than in ‘Fuding Dabaicha’ (Table S2).

### CsWRKY57like belongs to the IIc sub-group of the WRKY family

**Figure 2:**
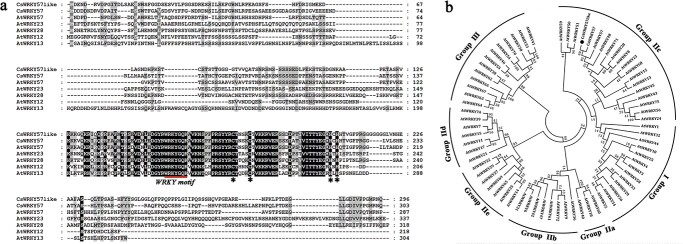
Amino acid sequence and phylogenetic relationship analysis of CsWRKY57like. (a) Multiple sequence alignment of CsWRKY57like with other WRKY proteins. Red lines and asterisks indicate the WRKY motifs and zinc-finger structures, respectively. (b) Phylogenetic analysis of CsWRKY57like and *Arabidopsis* WRKYs.

According to our RNA-seq and *Camellia sinensis* var. *sinensis* (CSS) (pcsb.ahau.edu.cn:8080/CSS/) databases, one full-length WRKY gene, designated *CsWRKY57like,* was upregulated in ‘Mingke 1’ and ‘Jinmudan’. The cDNA length of *CsWRKY57like* is 891 bp, and it encodes 296 amino acids. The molecular weight and pI values of CsWRKY57like are 32.05 kDa and 6.01, respectively. Analysis of its amino acid sequence suggested that CsWRKY57like possesses a highly conserved WRKY domain and a zinc-finger motif (C2H2 type) at the C terminus (Figure 2a).

The WRKY family can be divided into three major groups (I–III) based mainly on the number of conserved WRKY domains and the type of zinc finger structure. Group II is further divided into five subclasses (IIa–IIe). Phylogenetic analysis showed that CsWRKY57like is an IIc sub-group member, along with AtWRKY48, AtWRKY23, and AtWRKY12 (Figure 2b). AtWRKY23 and AtWRKY12 are capable of regulating plant secondary metabolism [23], suggesting the possible involvement of CsWRKY57like in the regulation of secondary metabolism in tea plants.

### Analysis of *CsWRKY57like* expression and sub-cellular localization

Similar to the FPKM expression data, RT-qPCR analysis showed that the transcript levels of CsWRKY57like were significantly higher in ‘Jinmudan’ and ‘Mingke 1’ than in the control cultivar ‘Fuding Dabaicha’ (Figure 1c), consistent with the EGCG3′′Me contents of these cultivars. These results imply that CsWRKY57like may positively regulate methylated EGCG biosynthesis in tea plants.

**Figure 3:**
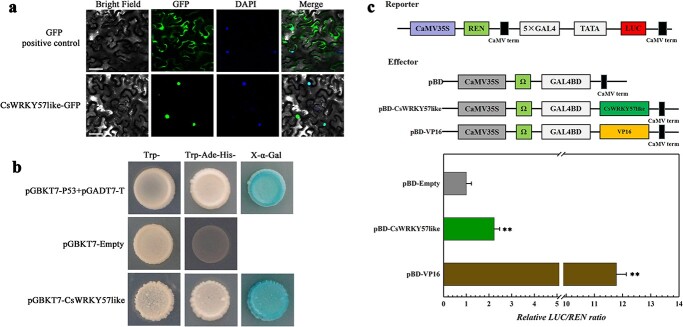
Characterization of CsWRKY57like. (a) Analysis of subcellular localization of CsWRKY57like in tobacco. Bars, 25 μm. (b) Transcriptional activities of CsWRKY57like in yeast cells. (c) The transcriptional activity of CsWRKY57like in *Nicotiana benthamiana* leaves. The pBD-Empty vector and pBD-VP16 served as the negative and positive control, respectively. **represents a significant difference (*P* < 0.01) compared with pBD-Empty and pBD-VP16.

Transcriptional regulation usually occurs in the nucleus. To explore the subcellular localization of CsWRKY57like *in vivo*, the coding region of CsWRKY57like was inserted into a GFP vector, and the GFP-Empty plasmid was used as the control. Both CsWRKY57like-GFP and the control plasmid were transiently expressed in tobacco leaves. As shown in Figure 3a, a GFP signal was observed throughout whole cells expressing GFP-Empty control, whereas CsWRKY57like-GFP was expressed only in the nucleus. This result implies that CsWRKY57like is a nuclear protein, which is characteristic of a typical TF.

**Figure 4:**
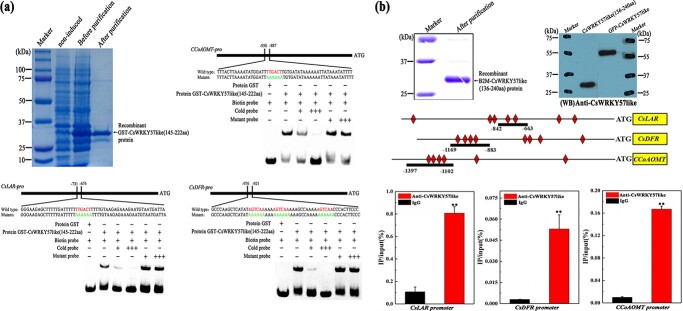
CsWRKY57like binds to the W-box element of *CsLAR-pro*, *CsDFR-pro*, and *CCoAOMT-pro in vitro* and *in vivo*. (a) *In vitro* binding of CsWRKY57like to *CsLAR-pro*, *CsDFR-pro*, and *CCoAOMT-pro* assessed by EMSA. (b) *In vivo* binding of CsWRKY57like to *CsLAR-pro*, *CsDFR-pro*, and *CCoAOMT-pro* assessed by ChIP-PCR analysis. ^**^P < 0.01.

### CsWRKY57like possesses trans-activation ability

To investigate whether CsWRKY57like possesses trans-activation activity, we analyzed the transcriptional activity of CsWRKY57like in yeast cells and tobacco. The coding region of *CsWRKY57like* was inserted into pGBKT7. As shown in Figure 3b, the yeast expressing pGBKT7-CsWRKY57like grew well on SD plates (−Trp-His-Ade), as did the positive control (p53 + T-antigen). This yeast-based assay indicates that CsWRKY57like possesses trans-activation ability.

A dual-luciferase reporter system was used to examine the transcriptional activity of CsWRKY57like in tobacco. The full-length sequence of *CsWRKY57like* was inserted into the pBD vector to generate pBD-CsWRKY57like, and the empty pBD and VP16 vectors were used as the negative and positive controls, respectively. As shown in Figure 3c, after expression in tobacco, both pBD-CsWRKY57like and VP16 showed a markedly higher LUC/REN ratio than the pBD-empty vector control (Figure 3c). This luciferase assay indicates that CsWRKY57like may act as a transcriptional activator.

### Three genes related to methylated EGCG biosynthesis are direct targets of CsWRKY57like

Based on the above findings, we wanted to understand the potential transcriptional regulatory mechanism by which CsWRKY57like affects methylated EGCG biosynthesis. Understanding whether CsWRKY57like can directly target genes involved in methylated EGCG biosynthesis might be interesting. Previous studies show that WRKY proteins directly target the W-box *cis*-elements in *CsLAR*, *CsDFR*, and *CCoAOMT* [9]. To explore whether CsWRKY57like could specifically recognize and directly bind to the promoters of genes related to methylated EGCG biosynthesis, an EMSA assay was performed. The probes covered about 50 bp that contained the *cis*-acting element (C/T)TGAC(T/C) in the predicted promoter sequence (Figure 4a). The coding region of *CsWRKY57like* (from amino acid position 145–222) was cloned into the pGEX-4T construct and transformed into *Escherichia coli* strain BL21(DE3). The CsWRKY57like recombinant protein was successfully expressed and purified (Figure 4a). An EMSA assay demonstrated that CsWRKY57like was capable of binding to the promoters of *CsLAR*, *CsDFR,* and *CCoAOMT* via the W-box motif, and the binding disappeared upon addition of excess unlabeled competitor probes. By contrast, GST protein could not bind sequences containing the *cis*-acting element (C/T)TGAC(T/C). This assay demonstrates that CsWRKY57like specifically binds to W-box *cis*-elements in the promoters of *CsLAR*, *CsDFR*, and *CCoAOMT*, indicating that genes involved in methylated EGCG biosynthesis are likely to be direct targets of CsWRKY57like.

The ability of CsWRKY57like to bind directly to target genes was further verified by an *in vivo* ChIP-qPCR assay using a polyclonal anti-CsWRKY57like antibody. As expected, compared with the IgG control, the *CsLAR*, *CsDFR*, and *CCoAOMT* promoter regions containing the *cis*-acting element (C/T)TGAC(T/C) were significantly enriched in anti-CsWRKY57like groups (Figure 4b). Taken together, these data illustrate that CsWRKY57like binds directly to the *cis*-acting element (C/T)TGAC(T/C) and directly targets methylated EGCG biosynthesis.

### CsWRKY57like regulates promoter activities of three genes related to methylated EGCG biosynthesis

**Figure 5:**
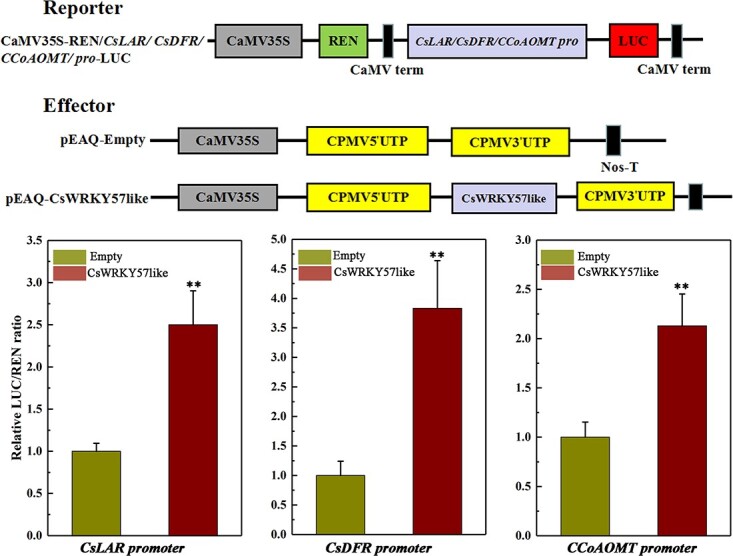
Dual-luciferase transient expression of CsWRKY57like regulates the expression of *CsLAR*, *CsDFR*, and *CCoAOMT*. **P < 0.01.

Dual-luciferase assays were performed to further understand the regulation of three genes related to methylated EGCG biosynthesis by CsWRKY57like. The promoter sequences of *CsLAR*, *CsDFR*, and *CCoAOMT* were cloned into the 0800-LUC plasmid, and CsWRKY57like was cloned into the PEAQ plasmid. These plasmids were transiently expressed in tobacco plants. As shown in Figure 5, CsWRKY57like showed a significantly higher LUC/REN ratio than the empty vector expressed together with the *CsLAR*, *CsDFR*, or *CCoAOMT* promoter. This result implies that CsWRKY57like is capable of activating the promoters of genes related to methylated EGCG biosynthesis.

### CsWRKY57like interacts with CsVQ4

**Figure 6:**
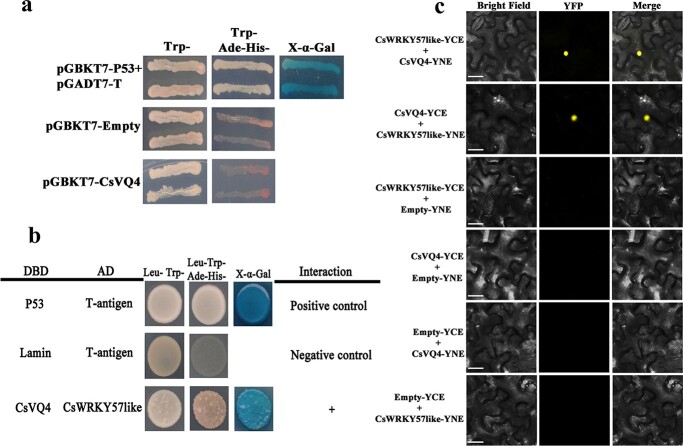
CsWRKY57like interacts with CsVQ4. (a) Transcriptional activities of CsVQ4 in yeast cells. (b) The interaction between CsWRKY57like and CsVQ4 by Y2H assay. (c) The interaction between CsWRKY57like and CsVQ4 in *N. benthamiana* leaf epidermal cells by bimolecular fluorescence complementation (BiFC) assay. Bar, 30 μm.

A large number of studies have shown that WRKY proteins can interact with different types of proteins, including VQ and MAPK proteins [24, 25]. To investigate whether CsWRKY57like might also interact with VQ motif-containing proteins, a Y2H library assay was performed. The candidate VQ motif-containing protein CsVQ4 was identified. As shown in Figure 6a and b, the yeast cells transformed with the negative control (pGBKT7-empty) and pGBKT7-CsVQ4 did not grow on SD plates, indicating that CsVQ4 showed no α-galactosidase activity (Figure 6a). Therefore, the full-length coding sequence of *CsVQ4* was cloned into the pGBKT7 vector, and *CsWRKY57like* was cloned into the pGADT7 vector for a Y2H assay. As shown in Figure 6b, the cells expressing BD-CsVQ4 + AD-CsWRKY57like grew well on SD plates (−Trp-His-Ade-Leu), as did the positive control (p53 + T-antigen). However, the negative control did not grow on SD plates (Figure 6b). This Y2H assay indicates that CsWRKY57like physically interacts with CsVQ4.

The interaction between CsWRKY57like and CsVQ4 was further analyzed by a BiFC assay. In this experiment, the coding regions of *CsWRKY57like* and *CsVQ4* were cloned into YCE and YNE vectors, respectively. As shown in Figure 6c, either CsWRKY57like-YCE and CsVQ4-YNE or CsWRKY57like-YNE and CsVQ4-YCE showed a strong yellow fluorescent signal in tobacco, unlike single fusion proteins, which produced no yellow fluorescent signal (Figure 6c). These results demonstrated that CsWRKY57like could interact with CsVQ4 in the nucleus (Figure S1) and may co-regulate the biosynthesis of methylated EGCG.

## Discussion and conclusions

**Figure 7:**
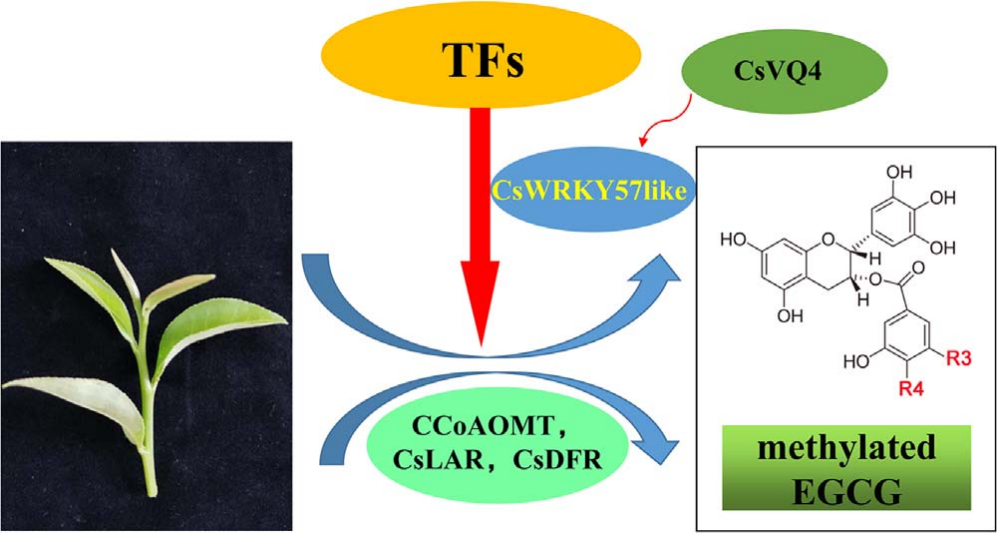
A model for the mechanism by which CsWRKY57like affects *O*-methylated catechin biosynthesis in tea plants (*Camellia sinensis* L.).

The WRKYs play indispensable roles in regulating plant physiological processes and are widely studied in plant stress resistance pathways [26, 27]. For instance, *Arabidopsis* WRKY52 was found to be involved in responses to disease and stresses [28]. CsWRKY2 was suggested to function in the drought stress response of tea plants [29]. Among the TF families, MYB and bHLH TFs have been extensively reported to participate in plant metabolic regulation [30, 31]. Recent studies have shown that WRKY TFs regulate several plant secondary metabolites, including flavonoids, alkaloids, and other substances [23, 32, 33]. Previous studies have shown that the biosynthesis of tea flavonoids is jointly regulated by structural genes and regulatory factors. In terms of methylated EGCG biosynthesis, the relevant structural genes have already been explored. However, the involvement of WRKY TFs in promoting the biosynthesis of methylated EGCG has been unclear. Therefore, we performed a systematic study of WRKY transcription factors in the tea plant (Figure 7).

In the present research, the WRKY subfamily IIc TF CsWRKY57like was identified from tea. We found that the expression of CsWRKY57like correlated well with the elevated accumulation of EGCG3′′Me (Figure 1). This finding prompted us to further investigate the function of CsWRKY57like in the biosynthesis of methylated EGCG in tea plants. A phylogenetic analysis showed that CsWRKY57like is clustered in the same clade with AtWRKY12 and AtWRKY23 of the *Arabidopsis* IIc subfamily (Figure 2). AtWRKY12 and AtWRKY23 belong to the same family and are involved in regulating the biosynthesis of lignin and flavonoids [32, 34]. Proteins of the same family usually have similar functions [35], suggesting that CsWRKY57like may regulate the biosynthesis of secondary metabolites in tea plants. However, it should be pointed out that studying the protein level of CsWRKY57like will be important in the future.

In general, TFs can only play their transcriptional regulatory role in the nucleus. Our assay showed that CsWRKY57like is a nuclear protein (Figure 3a), which is consistent with the nuclear localization of TFs [36]. WRKY TFs can act as either transcriptional activators or transcriptional repressors to activate or inhibit target gene expression and regulate different biological processes [37, 38]. For example, WSR1 is a novel WRKY transcription factor in *Brassica napus* that regulates leaf senescence by activating the expression of *senescence-associated gene 14* [39]. In this study, we provided evidence that CsWRKY57like controls the biosynthesis of methylated EGCG by regulating the transcription of three genes related to methylated EGCG biosynthesis: *CsLAR*, *CsDFR*, and *CCoAOMT*. We found that CsWRKY57like acts as a transcriptional activator (Figure 3b and c) and can specifically bind to the *cis*-acting element (C/T)TGAC(T/C) in the *CsLAR*, *CsDFR*, and *CCoAOMT* promoters (Figure 4), activating their transcription. This indicates that CsWRKY57like may positively regulate EGCG3′′Me biosynthesis (Figure 5). Taken together, our results provide new evidence about how WRKY TFs regulate methylated EGCG biosynthesis and are consistent with previous research suggesting that WRKYs may regulate the biosynthesis of catechins, L-theanine, and caffeine in tea plants [40, 41].

Recently, flavonoid biosynthesis was shown to be controlled by transcriptional complexes such as MYB–bHLH–WD40 (MBW) in tea plants. Thus, MYB was capable of interacting with bHLH and WD40 to co-regulate the biosynthesis of important secondary metabolites [35, 42]. Similarly, extensive studies have established that WRKY TFs, particularly subfamily I or IIc WRKYs, interact with several different types of proteins, such as VQ proteins [43, 44]. In the current study, our Y2H and BiFC assays revealed that CsWRKY57like physically interacts with the VQ protein CsVQ4 (Figure 6). This finding demonstrates that CsVQ4 may collaboratively regulate the biosynthesis of EGCG3′′Me. In general, interaction with VQs may differentially affect the properties and DNA-binding activities of the interacting WRKYs. For instance, *Arabidopsis* WRKY33 can interact with two VQ motif–containing proteins to enhance its DNA binding activity [45]. Therefore, it would be interesting to investigate whether CsVQ4 could repress or activate the properties of CsWRKY57like in future studies. In addition, the biosynthesis of catechins is controlled by many TFs and is believed to be a complicated biological process [46]. Therefore, identifying and dissecting other regulators, such as MYBs, bHLHs, and MADS, as well as their functions associated with methylated catechin biosynthesis, will be interesting and important for future research. Therefore, our findings have shed light on the regulatory network of methylated EGCG biosynthesis.

In summary, we demonstrated that the WRKY TF CsWRKY57like from *C. sinensis* may positively regulate methylated EGCG biosynthesis. CsWRKY57like acts as a transcriptional activator of three genes involved in methylated EGCG biosynthesis by specifically interacting with their promoters. Furthermore, CsWRKY57like may physically interact with CsVQ4. We propose that CsWRKY57like collaborates with CsVQ4 and participates in the metabolic regulation of methylated catechin biosynthesis (Figure 7). Taken together, our findings shed light on the regulatory network of catechin biosynthesis, as well as the transcriptional regulatory mechanism of secondary metabolism in tea plants. They suggest a potential strategy for the breeding of specific tea cultivars with high methylated EGCG levels.

## Materials and methods

### Plant materials

The three tea cultivars ‘Jinmudan’, ‘Mingke 1’, and ‘Fuding Dabaicha’ were grown at the experimental station of Gao Qiao tea garden, Changsha, Hunan, China. Fresh tea leaves (two leaves and a bud) were obtained from each cultivar, and three biological replicates were taken for each sample. The samples were stored at −80°C for further use after flash freezing in liquid nitrogen.

### cDNA synthesis, sequencing, and real-time PCR analysis

Total RNA was isolated from each frozen tea leaf sample using the RNeasy Mini kit (Qiagen, Hilden, Germany) according to the manufacturer’s protocol. RNA quantity and purity were measured by 1.0% agarose gel electrophoresis and spectrophotometry. Genomic DNA was removed from the total extracted RNA, which was then reverse-transcribed into first-strand cDNA following the manufacturer’s protocol.

According to our transcriptome FPKM expression data related to catechins and EGCG3′′Me biosynthesis, the upregulated *WRKY* gene *CsWRKY57like* was identified and selected. The full-length coding region of *CsWRKY57like* was amplified and confirmed by sequencing. The gene sequence was blasted at NCBI to identify homologous sequences. GeneDoc and ClustalX software were used for sequence alignment, and MEGA5 software was used for phylogenetic tree construction. The primers used are described in Table S1.

qRT-PCR was performed to measure gene expression levels. The reactions were performed in a total volume of 20 μL, and the PCR conditions were as described in our previous study [9, 47]. The relative gene expression levels were calculated using the formula 2^−ΔΔCt^. The primers are described in Table S1.

### Subcellular localization

To study the subcellular localization of *CsWRKY57like,* it was transiently expressed in tobacco. The full length of *CsWRKY57like* was cloned into a GFP vector. The primers are described in Table S1. 35S-*CsWRKY57like*-GFP and control 35S-GFP were transiently transformed into 1-month-old tobacco leaves as described previously [9, 48]. Fluorescent images were obtained with a Zeiss Axioskop 2 Plus microscope after 2–3 days of transient expression.

### Transcriptional activation in yeast cells

Transcriptional activities of *CsWRKY57like* and *CsVQ4* were assayed in yeast cells. The full-length sequences of the genes were independently cloned into the pGBKT7 vector (Clontech, USA). Primer sequences are shown in Table S1. All constructs were separately transformed into yeast cells and plated on different media (SD/−Trp or SD/−Trp-His-Ade), as the transcriptional activities of *CsWRKY57like* and *CsVQ4* depend on the growth status and *α*-galactosidase activity of yeast cells. Three biological replicates were performed for all transcriptional activation assays.

### Protein purification and EMSA assay

The cDNA sequence of *CsWRKY57like* (from amino acid position 145–222) covering the WRKY protein domain was cloned into the pGEX-4T vector (Amersham Biosciences) and expressed in *E. coli* strain BM Rosetta (DE3). The quality of the recombinant CsWRKY57like protein was estimated by SDS−PAGE, and it was then stored at −80°C for the EMSA assay. The primers are described in Table S1.

Probes including the *cis*-acting element (C/T)TGAC(T/C) from the promoters of *CsLAR*, *CsDFR*, and *CCoAOMT* were labeled with biotin. The EMSA experiment was performed as described previously [48, 49]. In brief, the assay mixtures of CsWRKY57like protein and biotin-labeled probes were incubated together, and the DNA–CsWRKY57like protein complexes were then analyzed by 6% native PAGE according to the manufacturer’s protocol.

### *CsWRKY57like*-specific antibody production and ChIP-qPCR analysis

The coding region of *CsWRKY57like* (from amino acid position 136–240) was cloned into the pET-B2M construct and transformed into *E. coli* strain BL21(DE3). The recombinant CsWRKY57like protein was induced, affinity purified, and separated by SDS–PAGE. Antibodies to CsWRKY57like were produced by Jinkairui Biotechnology Company (Wuhan, China). The ChIP-qPCR assay was performed as described by Fan et al. [49]. In brief, fresh tea leaves were crosslinked in 1% formaldehyde and then neutralized with glycine (0.125 M). The chromatin was pretreated by sonication and sheared to an average length of 500 bp. Immunoprecipitation was performed using a specific anti-CsWRKY57like antibody. The pre-immune serum IgG was used as the negative control. Protein A/agarose beads were used to capture the DNA–protein–antibody complex for 1 h at 4°C, followed by pelleting and washing of the beads. The immunoprecipitated material was eluted by gently rotating the reverse crosslinking immunoprecipitated DNA. After treatment with proteinase K, the immunoprecipitated DNA was purified and eluted. The DNA immunoprecipitated by the anti-CsWRKY57like antibody was amplified by qRT-PCR. The percentage of IP/Input was calculated by determining 2^−ΔCt^ (=2^−[Ct(IP)−Ct(Input)]^). The primers are described in Table S1.

### Dual-luciferase reporter assay.

To assess the transcriptional activity of CsWRKY57like in tobacco, the full-length coding sequence of *CsWRKY57like* was cloned into the pBD vector. The pBD vector is a double-reporter vector that contains a GAL4-LUC and an internal control REN, as described previously [48, 49]. VP16 was used as a positive control.

To assess the interaction of CsWRKY57like with the promoters of genes related to methylated EGCG biosynthesis, the *CsWRKY57like* coding region was cloned into the pEAQ vector as an effector, and the promoters of *CsLAR*, *CsDFR*, and *CCoAOMT* were ligated into the 0800-LUC vector as reporters. The primers are described in Table S1. *Agrobacterium* EHA105 containing all the constructs was transformed into tobacco. LUC and REN luciferase were analyzed after 3 days of infiltration using a dual-luciferase assay kit (Promega, USA). The transactivation ability of CsWRKY57like was indicated by the luciferase activity.

### Y2H and BiFC assay

For the Y2H assay, full-length coding sequences of *CsWRKY57like* and *CsVQ4* were cloned into pGADT7 and pGBKT7 vectors, respectively. The primers are described in Table S1. After testing for self-activation ability, all constructs were introduced into yeast strain Y2HGold for interaction assays according to the manufacturer’s instructions. Interaction assays were performed on different media (SD/−Trp-Leu or SD/−Trp-His-Ade-Leu), and the yeast growth status and *α*-galactosidase activity were measured. Three biological replicates were performed.

Full-length coding sequences of *CsWRKY57like* and *CsVQ4* were separately cloned into the yellow fluorescent protein (YFP) vectors *pSPYCE* and *pSPYNE*, respectively. The primers are described in Table S1. *Agrobacterium* EHA105 containing all the constructs was transformed into tobacco. YFP fluorescent images were obtained under a fluorescence microscope.

### Statistical analyses

All assays were performed with three biological replicates. All data in this research are presented as Mean ± S.E. The statistical significance of differences between means was tested by Student’s *t*-test, and *p* < 0.05 or *p* < 0.01 were considered significant.

## Acknowledgements

This work was funded by the National Natural Science Foundation of China (31470692), the Natural Science Foundation of Hunan Province (2020JJ5525), and the Research Foundation of the Education Bureau of Hunan Province (19C1720).

## Author contributions

Y.L., K.W.,J.H., and Z.L. conceived the research and designed the experiments; Y.L., X.H., X.S., B.W., and N.X. performed the experiments; Y.L. and X.H. analyzed the data and wrote the manuscript with contributions from all authors.

## Data Availability Statement

All data, models, and code generated or used during the study appear in the submitted article.

## Conflict of interest

The authors declare no competing financial interest.

## Supplementary data

Supplementary data is available at *Horticulture Research* online.

## Supplementary Material

Web_Material_uhac024
