# Supplementary material for: Identification of a WRKY transcriptional activator from Camellia sinensis that regulates methylated EGCG biosynthesis
Source: Hortic Res. 2022 Feb 19;9:uhac024. doi: 10.1093/hr/uhac024 (PMC9071374; doi:10.1093/hr/uhac024)
Supplement: Web_Material_uhac024 [file web_material_uhac024.zip › 57likeSupplementary Information.doc]

**Supplementary Table S1.** Summary of primers used in this study

| **Assay** | **Primer sequence (5’-3’)** | **Restriction Site** |
| --- | --- | --- |
| **Full length cloning** | CsWRKY57like-F: ATGGATGAGAACGACAGAGTCG |  |
|  | CsWRKY57like-R: TCATTGATTGCGCATTCCGGGA |  |
|  | CsVQ4-F: ATGGAGGACACACCAAAAATCC |  |
|  | CsVQ4-R: TCAAATACCCGAAACTCTTGGAG |  |
| **qRT-PCR** | CsWRKY57like -F: GTTCGGAGGTAGCGACAGAG |  |
|  | CsWRKY57like -R: ATCCTCCTCCATACCGATCC |  |
|  | β-actin-F: GCCATCTTTGATTGGAATGG |  |
|  | β-actin-R: GGTGCCACAACCTTGATCTT |  |
| **Subcellular localization** | CsWRKY57like -GFP-F: ttctgcccaaatcgcg ATGGATGAGAACGACAGAGTCG | ***Nru I*** |
|  | CsWRKY57like -GFP-R: tagtcataccggtcgc TTGATTGCGCATTCCGGGA | ***Nru I*** |
|  | CsVQ4-GFP-F:ttctgcccaaatcgcg ATGGAGGACACACCAAAAATCC | ***Nru I*** |
|  | CsVQ4-GFP-R: tagtcataccggtcgc AATACCCGAAACTCTTGGAG | ***Nru I*** |
| **Y2H assay** | CsWRKY57like -BD-F: Catggaggccgaattc ATGGATGAGAACGACAGAGTCG | ***EcoRI*** |
|  | CsWRKY57like -BD-R: Gccgctgcaggtcgacg TCATTGATTGCGCATTCCGGGA | ***SalI*** |
|  | CsWRKY57like -AD-F: GGAGGCCAGTgaattc ATGGATGAGAACGACAGAGTCG | *EcoR* I |
|  | CsWRKY57like -AD-R: CGAGCTCGATggatccTCATTGATTGCGCATTCCGGGA | *BamH* I |
|  | CsVQ4-BD-F: Catggaggccgaattc ATGGAGGACACACCAAAAATCC | ***EcoRI*** |
|  | CsVQ-BD-R: Gccgctgcaggtcgacg TCAAATACCCGAAACTCTTGGAG | ***SalI*** |
|  | CsVQ4-AD-F: GGAGGCCAGTgaattc ATGGAGGACACACCAAAAATCC | *EcoR* I |
|  | CsVQ-AD-R: CGAGCTCGATggatcc TCAAATACCCGAAACTCTTGGAG | *BamH* I |
| **BiFC assay** | CsWRKY57like-YC/NE-F: cgccactagtggatcc ATGGATGAGAACGACAGAGTCG | ***BamH I*** |
|  | CsWRKY57like-YC/NE -R: TAGCCTCGAGgtcgac TTGATTGCGCATTCCGGGA | ***Sal I*** |
|  | CsVQ4-YC/NE -F: cgccactagtggatcc ATGGAGGACACACCAAAAATCC | ***BamH I*** |
|  | CsVQ4-YC/NE -R: TAGCCTCGAGgtcgac AATACCCGAAACTCTTGGAG | ***Sal I*** |
| **Dual-luciferase transient expression assay** | CsWRKY57like -pBD-F: tcgccgaccggtaggcct ATGGATGAGAACGACAGAGTCG | ***Stu I*** |
|  | CsWRKY57like -pBD-R: aaccagagttaaaggcct TTGATTGCGCATTCCGGGA | ***Stu I*** |
|  | CsWRKY57like -pEAQ-F: caaattcgcgaccggt ATGGATGAGAACGACAGAGTCG | ***AgeI*** |
|  | CsWRKY57like -pEAQ-R: agttaaaggcctcgag TTGATTGCGCATTCCGGGA | ***XhoI*** |
|  | CsLAR-pro-LUC-F: Tatagggcgaattgg GCGTGTAACTAGTGTATTATGGGC | ***KpnI*** |
|  | CsLAR-pro-LUC-R: Ttggcgtcttccatgg TCTTTTCGTTACGTACCTCCCTC | ***NcoI*** |
|  | CsDFR-pro-LUC-F: Tatagggcgaattgg TTCCACCAATCCTGCAATCATAGGA | ***KpnI*** |
|  | CsDFR-pro-LUC-R: Ttggcgtcttccatgg GATGGGATTAAAATGTGAAAAAA | ***NcoI*** |
|  | CCoAOMT-pro-LUC-F: Tatagggcgaattgg GCGTGTCTGCCTCTTCATCATG | ***KpnI*** |
|  | CCoAOMT-pro-LUC-R: Ttggcgtcttccatgg TATTGCAACGAATTAATTCTGTGT | ***NcoI*** |
| **EMSA assay** | CsWRKY57like(145-222aa)-GST-F:ggttccgcgtggatcc AGCGAAGTTGATCATCTTGAAGATG | ***BamHI*** |
|  | CsWRKY57like(145-222aa)-GST-R: agtcacgatgcggccgc TCATCCACCACCTCCACCTC | ***NotI*** |

**Supplementary Table S2.** Transcript abundance of *CsWRKY57like* in the “Jinmudan vs Fudingdabai” and “Jinguanyin vs Fudingdabai” pairwise comparison

| Gene  name | Gene_id | Jinmudan vs Fudingdabai | | Jinguanyin vs Fudingdabai | |
| --- | --- | --- | --- | --- | --- |
|  |  | FPKM | Log2FC | FPKM | Log2FC |
| CsWRKY57like | CSA014188 | 11.66/4.80 | 1.18 | 7.37/4.80 | 0.68 |

**Supplementary Text S1.** Nucleotide sequence of *CsWRKY57like*.

***>*** ***CsWRKY57like***

ATGGATGAGAACGACAGAGTCGATCCAGGAATTACAGATTTATCGGCAGAGTCGAGCTGGTCGTTCGGCGGCGACTCGGAGAGCGTTTACTTGTTCGGAGGTAGCGACAGAGAGAGCAGCATACTCAGCGAATTCGGCTGGAATCTCCGGCCAGAGGCAGGTAGTTTTTCTCACTTTGATCGGATCGGTATGGAGGAGGATTTGGCCGGAAATGATCACCCGAAAAGTACTTGTTCTACATCTACCACTGGTGGGGGTGGGACGGTGGTTCAAGCGACGTCGAATCGGTCGATGTCTTCGAGCTCCTCCGAGGATCTGCCGGAGAAGTCGACGGAGTCCGACGGGAACAAAGCAGCGCCTGAGACAGCGAGCAAAGTAAAAAAGAAGGGGCAAAAGAGAATCCAACAGCCACGTTTTGCATTCATGACTAGGAGCGAAGTTGATCATCTTGAAGATGGTTATCGATGGCGGAAATATGGCCAGAAAGCAGTTAAAAATAGTCCATTTCCTAGGAGCTATTATCGGTGTACAAACAGCAAATGCACAGTGAAGAAAAGGGTTGAACGCTCTTCAGATGATCCAACTATTGTAATCACTACATACGAAGGCCAGCATTGTCACCATACAGTTGGATTCCCCAGAGGTGGAGGTGGAGGTGGTGGACTCGTCAATCATGAAGCTGCCTTCGCAAGCCAATTGACTCCTTCAGCTTCACATTTTTATTATTCAGGACTAGGAGGACTCCAATTTCCTCCACAACCTCCTCCTCTCGGTATTATACCACAATCATCATCTCATCAAGTTCCAGGTGAAGCAAGAGAAAATCCTAAGCTTCCCACTGATGAAGGACTTCTCGGTGACATTGTACCTCCCGGAATGCGCAATCAATGA

**Supplementary Figure S1.** Subcellular localization of CsVQ4 was transient expressed in Nicotiana benthamiana leaves. Bars, 25 μm.
